# Extragonadal mixed germ cell tumor of the right arm: description of the first case in the literature

**DOI:** 10.1186/1477-7819-10-69

**Published:** 2012-05-19

**Authors:** Hicham Ait Benali, lssam Lalya, Mohamed Allaoui, Aniss Benmansour, Basma Elkhanoussi, Samir Benjelloun, Noureddine Benjaafar, Mourad Elyaacoubi

**Affiliations:** 1 Department of Trauma Surgery, Teaching Hospital Avicenne, Mohamed V University, Rabat, Morocco; 2 Department of Radiation Oncology, National Institute of Oncology, Mohamed V University, Rabat, Morocco; 3 Department of Pathology, National Institute of Oncology, Mohamed V University, Rabat, Morocco; 4 Department of Surgical Oncology, National Institute of Oncology, Mohamed V University, Rabat, Morocco

**Keywords:** Chemotherapy, Embryonic carcinoma, Extragonadal, Mixed germ cell tumors, Surgery, Teratoma

## Abstract

**Background:**

Extragonadal localization of germ cell tumors (GCTs) is rare; to the best of our knowledge, a location in the soft tissue of the arm has never been previously reported in the literature.

**Case presentation:**

We report the case of a 37-year-old man who presented with a primary malignant mixed non-seminomatous GCT (teratocarcinoma variety) in the right arm, treated by a combination of cisplatin-based chemotherapy and surgery. After 18 months of close follow-up, no locoregional recurrence or distant metastases have been detected.

**Conclusions:**

A combination of chemotherapy and surgery is the most appropriate treatment strategy for extragonadal GCTs, to ensure both local and systemic control.

## Background

Germ cell tumors (GCTs) are classified as extragonadal if there is no evidence of a primary tumor in either the testes or ovaries [1]; they typically arise in midline locations. In adults, the most common sites, in order of frequency, are the anterior mediastinum, retroperitoneum, and the pineal and suprasellar regions. To date, there have been no reported cases of germ cell tumors arising in the arm.

The aim of the present work was not only to report what is to the best of our knowledge the first observation of a mixed non-seminomatous GCT of the arm, but to also increase exposure of the various hypotheses that could explain this unusual location. We also discuss the diagnosis, treatment, and prognosis of this entity.

## Case presentation

**Figure 1:**
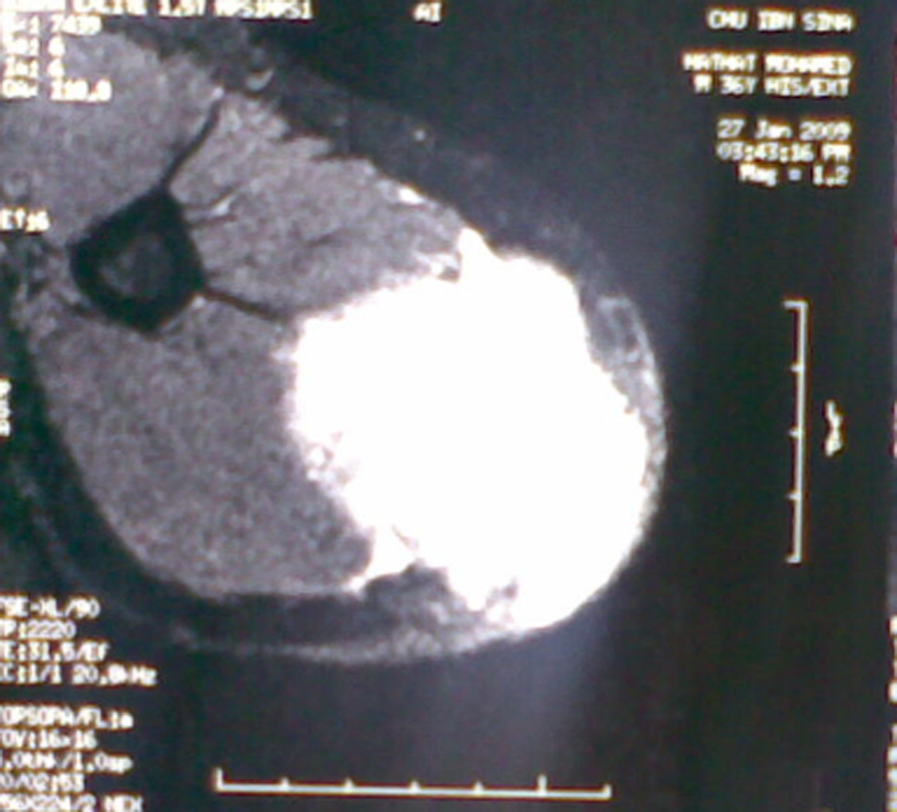
MRI image after administration of gadolinium-diethylenetriaminepentacetate (Gd-DTPA) axial showing a 53 × 40 mm diameter soft tissue mass in the posterior compartment of the right arm.

**Figure 2:**
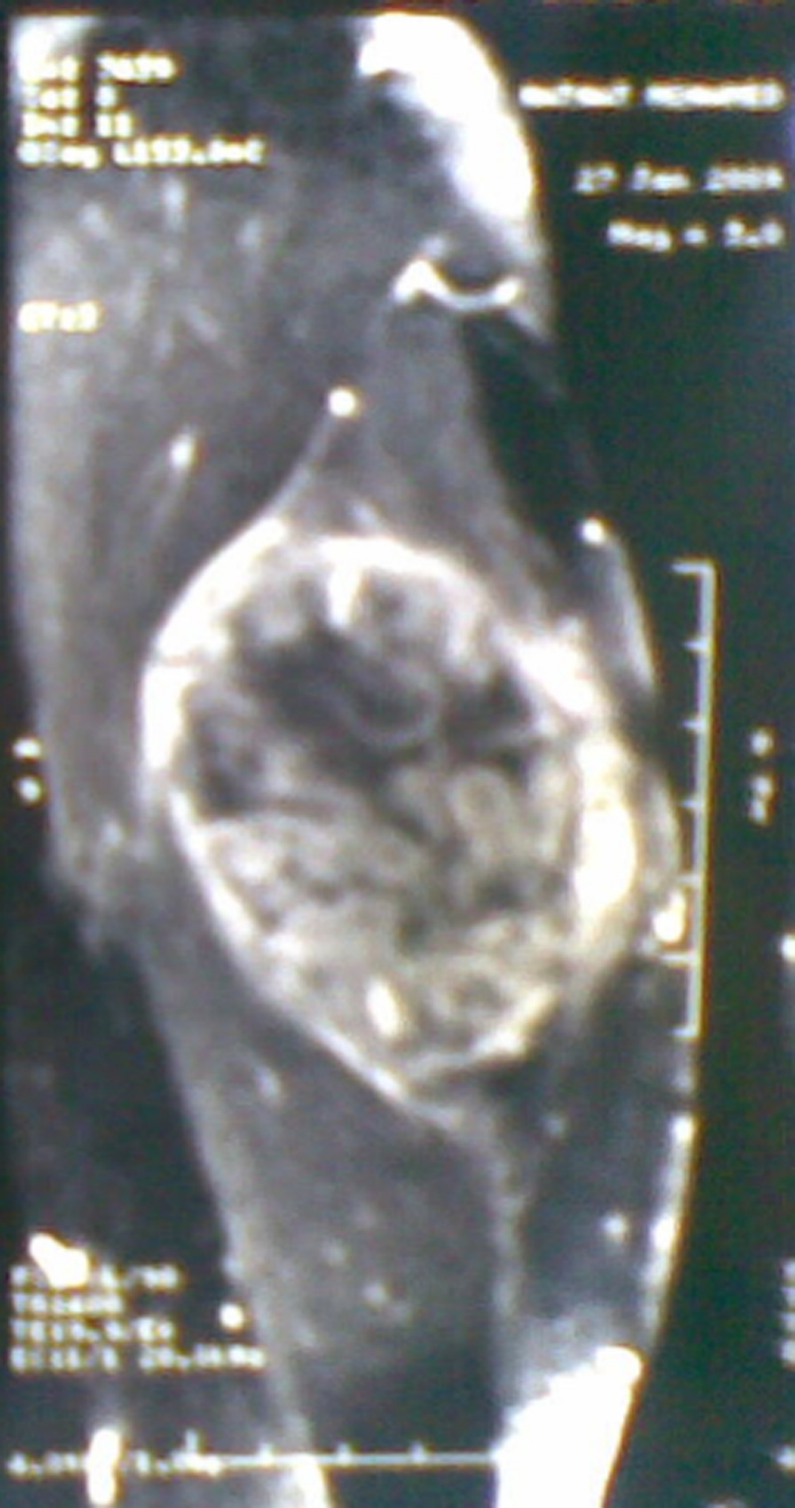
MRI image after administration of gadolinium-diethylenetriaminepentacetate (Gd-DTPA) (coronal) showing a 53 × 40 mm diameter soft tissue mass in the posterior compartment of the right arm.

**Figure 3:**
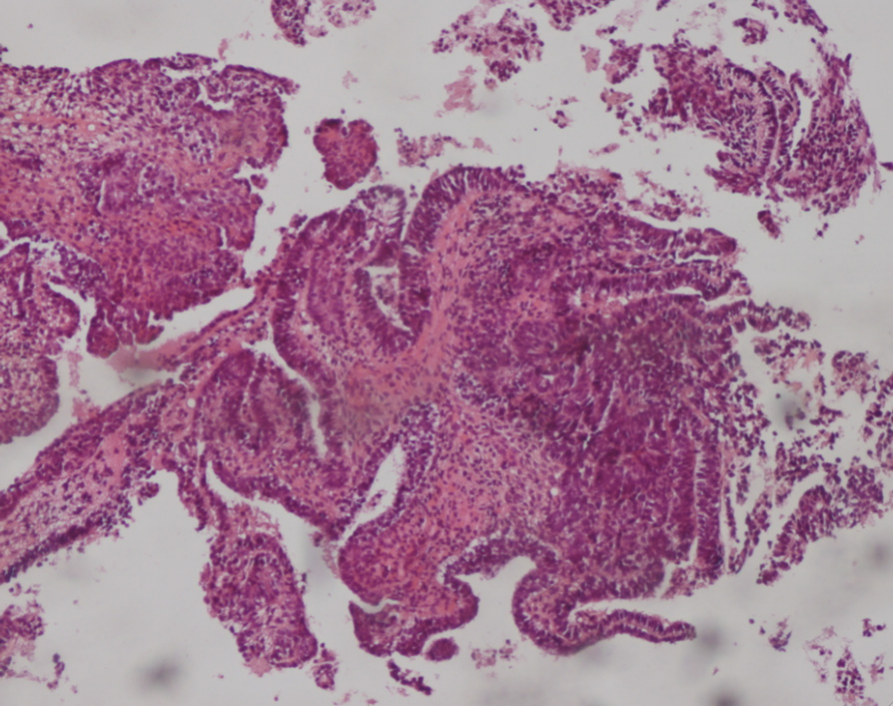
Different epithelial structures associated to sarcomatous component (hematoxylin and eosin stain, 40 ×).

**Figure 4:**
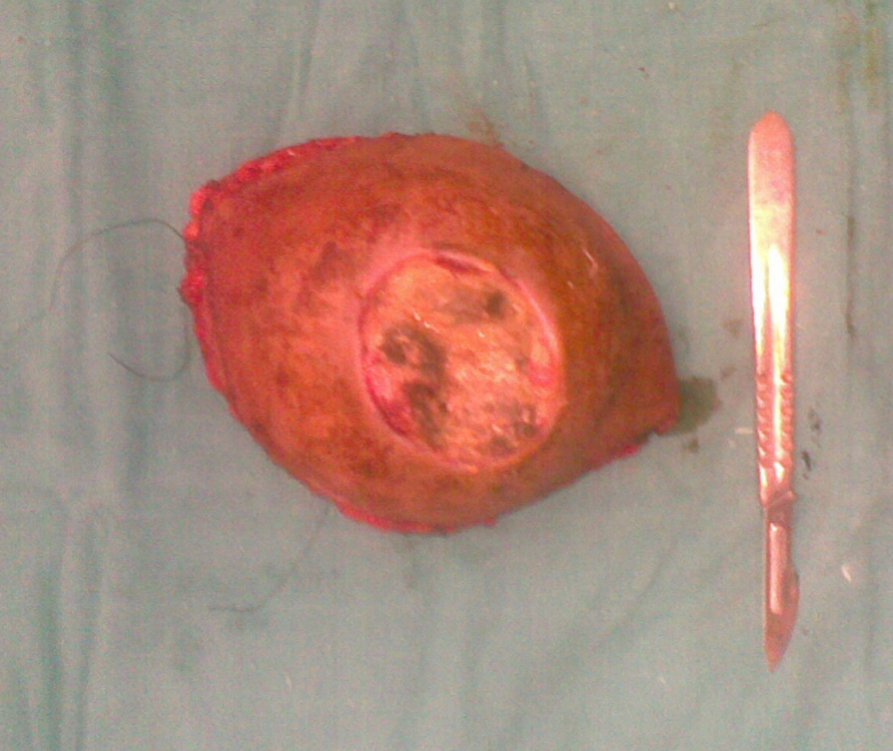
Macroscopic aspect.

**Figure 5:**
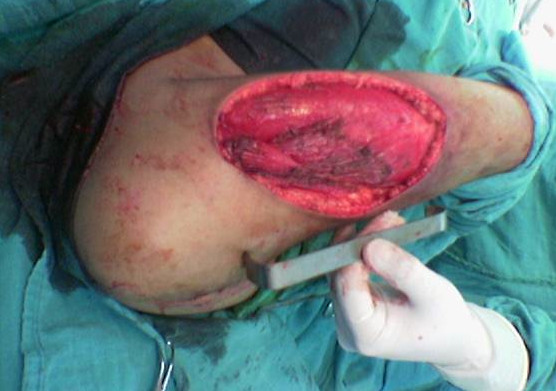
Tumor bed.

A 37-year-old man presented with a 2-year history of painless swelling of the right arm with a gradual increase in size. A physical examination was normal except for a well circumscribed non-tender mass in the upper two-thirds of the right arm and multiple lymph nodes in the right axilla. Imaging revealed a 53 × 40 mm diameter soft tissue mass that was hyperintense on T2-weighted MRI in the posterior compartment of the right arm, with no bone or vascular invasion (Figures 1 and 2). Surgical biopsy of the mass and axillary lymph node excision were performed; the laboratory received two fragments with soft consistency, measuring 2 × 1 × 0.3 cm and 2.5 × 1 × 0.5 cm. Histopathological examination showed a desembryoplastic multitissular tumor, containing a sarcomatous component constituted by spindle cells and organized in bundles with rhabdomyoblastic differentiation. There was also an immature and malignant neuroglial component, and this tumor additionally showed epithelial structures with squamous or glandular differentiation; some cells were compatible with embryonal carcinoma. These various tissular structures were very confluent, without transition. Atypical immature cartilage and bone components were observed associated with necrosis. The axillary lymph node excised was metastatic. There was no need for immunohistochemical staining to confirm the diagnosis of malignant mixed GCT (teratocarcinoma variety) (Figure 3). Testicular palpation and ultrasonography results were normal. A computed tomography (CT) scan of the chest, abdomen and pelvis showed no abnormalities. Serum α-fetoprotein (AFP), β-human chorionic gonadotropin (β-HCG), and serum lactate dehydrogenase (LDH) levels were all within normal ranges. The patient received four courses of chemotherapy (bleomycin 30 units intravenous injection, days 1, 8, and 15; etoposide 100 mg/m^2^ intravenously, days 1 through 5; cisplatin 20 mg/m^2^ intravenously, days 1 through 5). A clinical evaluation of response at the end of chemotherapy showed a stable disease. Then, 1 month later, a wide excision with axillary dissection was performed; the excised tumor was partially well circumscribed and measured 57 × 42 × 38 mm and had a uniform, yellowish, solid, and partially nodular appearance on the cut surface. Final pathology revealed the same histological aspect as observed in the biopsy but did not revealed tumoral necrotic patterns. All surgical margins were free, and one of six lymph nodes identified was involved without extracapsular spread (Figures 4 and 5). Two further cycles of chemotherapy using the same protocol were added. At 18 months of close follow-up, no locoregional recurrence or distant metastases have been detected.

## Discussion

Primary GCTs of extragonadal origin comprise 3% to 5% of all germ cell tumors. Extragonadal GCTs arise from midline structures [2,3]. A case of extragonadal malignant teratoma of the extremities has been reported by Chinoy *et al*. [4]. Herein, we describe what is to the best of our knowledge the first reported case of a mixed non-seminomatous germ cell tumor (teratocarcinoma) located in the soft tissue of the right arm. The histogenesis of extragonadal GCTs is not clearly defined: two competing hypotheses have been proposed, but there are inadequate data to determine which, if either, is correct. The first hypothesis is that extragonadal GCTs are derived from primordial germ cells that fail to complete the normal migration along the urogenital ridge to the gonadal ridges during embryonal development. This may be due to an abnormality in the primordial germ cell itself or in its microenvironment [5]. The second main hypothesis is that germ cells transformed in the testes undergo reverse migration [6]. This hypothesis is supported by genetic data suggesting that extragonadal GCTs and testicular GCTs share a common cell of origin [7,8]. The etiology of extragonadal GCTs is unknown; in rare cases, they have been associated with Klinefelter syndrome [9-12]. A biopsy is required for definitive diagnosis and treatment of extragonadal GCTs; the majority of patients have clear evidence of germ cell features or teratoma, while a small subset have a poorly differentiated tumor without distinctive germ cell features. In our patient’s case the diagnosis of teratocarcinoma was established on the basis of pathological examination, which showed the combination of teratoma and embryonal carcinoma [13]. However it is important to note that the term teratocarcinoma has largely been abandoned, and these tumors are referred to as a malignant mixed GCT, with a description of the specific germ cell tumor elements present. Once the diagnosis of a germ cell tumor has been established, a primary testicular tumor must be excluded. Testicular palpation is insufficient to exclude a testicular primary; ultrasonography should be performed in all patients [14]. It may be difficult to distinguish true extragonadal GCTs from metastatic tumors in which the primary testicular lesion has regressed [15-17]. Extragonadal non-seminomatous GCT should be considered in the differential diagnosis of histologically poorly differentiated cancer and of neoplasms of unknown primary site, particularly in young men with midline disease. In our patient’s case another uncommon differential diagnosis must be evocated: it is a soft tissue metastasis from primary testicular cancer [18].

Because an extragonadal GCT may be curable with cisplatin-based chemotherapy, many recommend that the diagnostic evaluation in such cases should include measurement of the serum tumor markers AFP and β-HCG, immunohistochemical assessment of the biopsy [19], and cytogenetic analysis for abnormalities of chromosome arm 12p [20]. However, isochromosome 12p is not pathognomonic of GCTs [21,22]. Mixed GCTs, mainly teratomas with additional malignant components (other germ cell tumors, carcinomas or sarcomas), have been reported to be more aggressive. More than 50% of those patients died within 2 years of follow-up due to local invasion or distant metastases (lymph nodes, liver, lung, heart, bone, and brain) [23,24]. A multimodality approach is generally used, utilizing chemotherapy initially followed by surgery for any residual mass. We recommend four cycles of bleomycin, etoposide and cisplatin (BEP) chemotherapy as the initial therapy, rather than surgery or radiation therapy; for patients with a residual mass following initial chemotherapy, we recommend complete surgical resection if technically feasible. If viable malignancy is identified, two additional cycles of chemotherapy should be given [25,26].

## Conclusions

Regardless of the location, the therapeutic approach of extragonadal GCTs should be multidisciplinary, combining systemic chemotherapy and surgery.

The decision of the number and timing of cycles of chemotherapy depends on the disease stage and histological analysis of the surgical specimen.

## Consent

Written informed consent was obtained from the patient for publication of this report. A copy of the written consent is available for review from the Editor-in-Chief of this journal.

## Misc

Hicham Ait Benali and lssam Lalya are equally contributed.

## Competing interests

The authors declare that they have no competing interests.

## Authors’ contributions

IL and HA performed the literature review, composed the case report and wrote the manuscript. MA and AB were involved with conception and design and collection and assembly of data. BEK and MA analyzed and interpreted the anatomopathology findings. SB, NB and ME approved the final manuscript. All authors read and approved the final manuscript.
